# Draft Genome Sequence of *Desulfurobacterium* sp. Strain AV08, a Thermophilic Chemolithoautotroph Isolated from a Deep-Sea Hydrothermal Vent

**DOI:** 10.1128/MRA.00615-21

**Published:** 2021-08-26

**Authors:** Emilie J. Skoog, Julie A. Huber, Margrethe H. Serres, Alice Levesque, Lisa Zeigler Allen

**Affiliations:** a Department of Earth, Atmospheric, and Planetary Sciences, Massachusetts Institute of Technology, Cambridge, Massachusetts, USA; b Department of Marine Chemistry and Geochemistry, Woods Hole Oceanographic Institution, Woods Hole, Massachusetts, USA; c Microbial and Environmental Genomics, J. Craig Venter Institute, San Diego, California, USA; d Marine Biology Research Division, Scripps Institution of Oceanography, La Jolla, California, USA; University of Southern California

## Abstract

A thermophilic chemolithoautotrophic bacterium was isolated from vent fluids at Axial Seamount, an active deep-sea volcano in the northeast Pacific Ocean. We present the draft genome sequence of *Desulfurobacterium* sp. strain AV08.

## ANNOUNCEMENT

Members of the *Desulfurobacterium* genus have been identified globally in deep-sea vents (1–6). *Desulfurobacterium* sp. strain AV08 was isolated from Bag City, a low-temperature (23°C) diffuse flow vent at Axial Seamount located on the Juan de Fuca Ridge (7). Fluid sampling was conducted using the hydrothermal fluid particle sampler (8) aboard the ROV *ROPOS*, at a depth of 1,520 m. Samples were immediately cultured anaerobically in Balch tubes by inoculation of 1 ml of fluid sample into 7 ml of artificial seawater medium with 0.001% yeast extract (9). Culture tubes were reduced with 0.05% Na_2_S and given a headspace of 80% H_2_/20% CO_2_. Cultures were incubated at 70°C. AV08 was then isolated by serial dilution to extinction (10) at 70°C.

After isolation, AV08 was grown in the laboratory at 70°C in *Sulfurimonas paralvinella* medium (DSMZ 1053 medium) reduced with 0.05% Na_2_S before inoculation and topped off with a headspace of 80% H_2_/20% CO_2_ after inoculation. Genomic DNA was extracted from a single culture using the cetyltrimethylammonium bromide (CTAB) phenol-chloroform-based extraction method and sequenced using both long-read (Oxford Nanopore Technologies [ONT]) and short-read (Illumina MiSeq) (PE 250 bp) sequencing technologies. Postprocessing, assembly, annotation, and bioinformatic analyses used default parameters for all software unless otherwise specified. The collective reads were assembled using a SPAdes v3.5.0 hybrid assembly (11) (Table 1). This resulted in 38 total contigs, with an *N*_50_ value of 354,053 bp, a maximum contig length of 815,718 bp, and a total length of 1,732,745 bp. CheckM v1.0.18 was used to determine genome completion and contamination values of 99.58% and 1.27%, respectively (12). QUAST v4.4 was used to identify a G+C content of 39.33% (13).

**TABLE 1:** Organism isolation, growth, and sequencing data

Parameter^*a*^	Data
Environmental data	
Geographic location	
Region	Juan de Fuca Ridge, northeast Pacific Ocean
Volcano	Axial Seamount
Geographic coordinates	45°58′N, 130°00′W
Collection date	July 1999
Marine biome	Hydrothermal vent
Vent name	Bag City
Vent temp (°C)	23
Sampling depth (m)	1,520
Sampling method	ROV-based fluid sampler
Growth conditions	DSMZ 1053 medium, anaerobically
Sequencing	
RNA/DNA quantification instrument	Nanodrop UV/visible spectrophotometer (Thermo Fisher Scientific)
Sample purity	
260 nm/280 nm	∼1.7
260 nm/230 nm	∼1.3
Nanopore library	ONT rapid PCR-free kit
Illumina library	Accel-NGS 2S Plus DNA library preparation kit/MiSeq nanokit v2
Sequencing technologies	ONT plus Illumina MiSeq (hybrid)^*b*^
No. of Nanopore reads	5,475
Nanopore read *N*_50_ (bp)	2,092
Longest Nanopore read length (bp)	80,194
No. of Illumina reads	736,493
Nanopore base calling and postprocessing	Guppy
Nanopore flow cell	ONT MinION R9.4.1
Sequencing center	J. Craig Venter Institute (La Jolla, CA)
Assembler	SPAdes v3.5.0
No. of contigs	38
Largest contig (bp)	815,718
Contig *N*_50_ (bp)	354,053
ORF caller	JGI IMG Annotation Pipeline
Genomic features	
Genome size (bp)	1,732,745
G+C content (mol%)	0.3933
No. of genes	1,824
No. of protein-coding genes	1,763
No. of genes with COG identity	1,472
No. of RNA genes	51
No. of rRNA genes	3
No. of 5S rRNA genes	1
No. of 16S rRNA genes	1
No. of 23S rRNA genes	1
No. of tRNA genes	46
No. of other RNA genes	2
No. of CRISPR loci	4

a ORF, open reading frame; COG, Clusters of Orthologous Groups of proteins.

b The same DNA extracted from a single culture was used for both library preparations and sequencing technologies.

**FIG 1:**
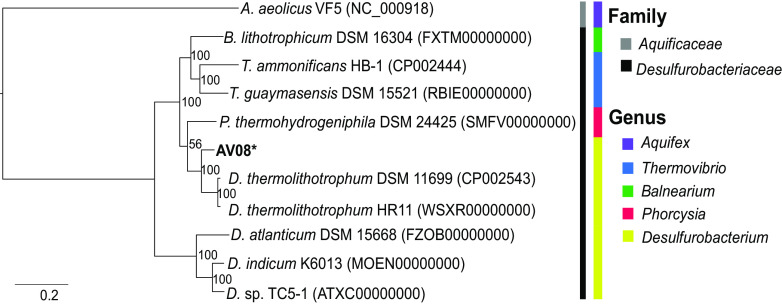
Phylogenomic tree of *Desulfurobacterium* sp. strain AV08 (marked with an asterisk) and related *Desulfurobacteriaceae* and *Aquificaceae* (outgroup) family strains. All available *Desulfurobacteriaceae* genomes with >97% genome completeness are included. Families and genera are indicated by color.

The genome was annotated with the Joint Genome Institute (JGI) Integrated Microbial Genomes (IMG) Annotation Pipeline v5.0.20 (14, 15), resulting in 1,763 protein-coding genes, 1 complete rRNA gene, 46 tRNA genes, and 4 CRISPR loci (Table 1). *Desulfurobacterium* sp. strain AV08 has genes for the reductive tricarboxylic acid (rTCA) cycle for CO_2_ fixation, sulfur reduction, and denitrification, as well as genes for hydrogen oxidation and formate utilization. The JGI IMG pipeline-annotated 16S rRNA gene was used to identify homologous sequences within the NCBI GenBank nonredundant database (16). Based on 16S rRNA gene analysis, AV08 is most similar to Desulfurobacterium crinifex NE1206 (GenBank accession number AJ507320.2) (3) (99.4%) and Desulfurobacterium thermolithotrophum DSM 11699 (GenBank accession number NR_075040.1) (6) (97.3%). A phylogenomic tree constructed with GToTree v1.4.5 (17) indicated that AV08 is most closely related to D. thermolithotrophum (Fig. 1). Currently, no genomic data are available for D. crinifex. Overall genome relatedness index values of 81.95 and 79.99 were determined using average nucleotide identity analyses within GTDB-Tk v1.1.0 (18) and the JGI IMG system, respectively. These values fall well below the 95% to 96% cutoff values for species determination.

### Data availability.

The complete genome sequence and metadata are publicly available at JGI IMG under analysis project number Ga0466268. Raw sequence reads are available at NCBI under BioProject accession number PRJNA731061 and SRA accession numbers SRR14730166 and SRR14730165.
